# Comparison of hip structure analysis and grip strength between femoral neck and basicervical fractures

**DOI:** 10.1186/s12891-021-04363-w

**Published:** 2021-05-19

**Authors:** Yong-Han Cha, Jun-Il Yoo

**Affiliations:** 1 Department of Orthopaedic Surgery, Eulji University hospital, Daejeon, South Korea; 2 Department of Orthopaedic Surgery, Gyeongsang national university hospital, Jinju, Gyeongnamdo South Korea

**Keywords:** Basicervical fracture, Hip fracture, Grip strength, Hip-structure analysis

## Abstract

**Background:**

The purpose of this study was to analyze differences in geometrical properties of the proximal femur and predict the occurrence of basicervical fractures through a comparative study of femoral neck and basicervical fractures in patients undergoing hip structural analysis (HSA).

**Methods:**

All patients with hip fractures who were at least 65 years old and admitted to our hospital between March 2017 and December 2019 were eligible for this study. During the study period, 149 femur neck fractures (FNF) and basicervical fractures (intertrochanteric fractures of A31.2) were included in this study. Fifty-nine patients were included in the final analysis. Factors considered to be important confounders affecting the occurrence of basicervical hip fractures were chosen for propensity-score analysis. A logistic model with basicervical hip fracture as the outcome and age, sex, weight, spinal T-score, hip T-score, and vitamin D levels as confounders was used to estimate the propensity score.

**Results:**

The cross-sectional moment of inertia **(**CSMI) of the intertrochanter was significantly lower in patients with basicervical hip fracture (HF) than in patients with FNF (*p* = 0.045). However, there was no significant differences in any other HSA variable between the two groups. Receiver operating characteristic (ROC) analysis showed that cutoff point for HSA was 100 for hip axis length (HAL) (AUC = 0.659, *p* < 0.001) and 5.712 for CSMI of the intertrochanter (AUC = 0.676, *p* < 0.001). ROC analysis showed that cutoff points of HAL, CSMI of intertrochanter, and handgrip strength were 104.8, 8.75, and 16.9, respectively (AUC = 0.726, *p* < 0.001).

**Conclusions:**

Proximal femoral geometric analysis using HSA is a useful method for predicting the type of hip fracture. Additionally, a lower CSMI, a shorter HAL, and a lower grip strength are major predictors of basicervical fractures.

## Introduction

Hip fractures occur in more than 250,000 people in the United States each year. They are estimated to double in 2050 [1, 2]. In particular, osteoporotic hip fractures are causing a socioeconomic problem worldwide [3, 4]. Hip fractures are classified anatomically as intracapsular fractures and extracapsular fractures, with femoral neck fractures and intertrochanteric fractures in each category [5]. Several factors such as demographic factors, the comorbidity of the patient, and fracture location are considered when determining the treatment method [6].

Basicervical fractures are defined as proximal femoral fractures located at the base of the femoral neck at its junction with the intertrochanteric region [7]. For this reason, they are classified as femoral neck fractures. However, from a therapeutic point of view, they are similar to intertrochanteric fractures because they are close to the intertrochanteric line [8]. Several studies have reported that failure rates of surgical treatment by internal fixation are high [7, 9]. Causes of such high failure rates have been explained by the anatomical location of the fracture, implant problems, and lack of rotational stability [10, 11]. However, previous studies lacked bone quality and geometrical analyses.

Understanding changes in bony structure and strength due to osteoporosis with an increasing age can be accomplished by analysis of geometric properties of bony tissues [12]. Understanding the pathogenesis of osteoporosis and hip fracture provides important information for the establishment of fracture treatment strategies. Bone mineral density measurements using dual-energy x-ray absorptiometry (DXA) can assess bone quantities. Geometrical properties can be evaluated using hip structural analysis (HSA), an output function of the DXA machine. Geometric parameters such as bucking ratio and intertrochanteric outer diameter can be used to predict incident hip fractures. Changes in mechanical structure of the femoral neck area have been reported to be associated with the risk of subsequent fractures of the contralateral hip [13, 14]. Previous studies have also reported that evaluating geometrical properties of femurs in hip fractures can help predict the risk of hip fractures [15, 16]. However, these studies only analyzed geometries of the proximal femur and the risk of fractures without analyzing characteristics according to the type of hip fractures.

Therefore, the purpose of this study was to analyze differences in geometrical properties of the proximal femur and predict the occurrence of basicervical fractures by comparing patients with femoral neck fractures and those with basicervical fractures undergoing HSA.

### Ethics statement

The design and protocol of this retrospective study were approved by the Institutional Review Board of our hospital (GNUH-2019-05-018-007). The requirement to obtain informed consent was waived by the board.

### Participants

All patients with a hip fracture who were at least 65 years old and admitted to our hospital between March 2017 and December 2019 were eligible for this study. During the study period, 149 femur neck and basicervical fractures (intertrochanteric fractures of A31.2) were included in this study. Basicervical fractures were defined as proximal femoral fractures located at the base of the femoral neck at its junction with the intertrochanteric region [7]. Because bed rest for several days after surgery could affect the skeletal mass index, there was concern about changes in body composition. Our hospital operates under a protocol in which hip fracture patients undergo DXA as soon as possible if there is no surgical delay. In this study, 16 (10.7%) patients who did not undergo DXA during their hospital stay were excluded. We were concerned about a decrease in skeletal mass index due to bed rest for several days after surgery. Thus, only patients who underwent DXA before surgery after admission were included in this study. Thus, 59 (39.6%) patients who underwent DXA after surgery were excluded. All operations were performed within 2 days after injury. Fifteen (10.1%) patients with mental health issues such as dementia, delirium, depression, and mental retardation were also excluded. Finally, 59 patients were included in this study for analysis.

### Biochemical analyses

Serum 25-hydroxyvitamin D (25[OH] vitamin D) levels were measured using a 1470 Wizard gamma counter (Perkin Elmer, Finland), an Automatic Analyzer 7600 (Hitachi, Japan), and LIAISON (DiaSorin, U.S.A.) for radioimmunoassay (25-hydroxyvitamin D ^125^ I RIA Kit; DiaSorin).

### Measurements of appendicular skeletal muscle mass, BMD, and handgrip strength

Body composition and bone mineral density (BMD) in both groups (HF and non-HF) were calculated based on DXA using a QDR 4500A apparatus (Hologic, U.S.A.). Bone mineral content, fat mass, and lean soft tissue mass were evaluated separately for each portion of the body, including arms and legs. The lean soft tissue mass of arms and legs was about equal to skeletal muscle mass. Since the absolute muscle mass corresponds to the height, SMI was determined as lean mass [kg]/height [m]^2^. Arm SMI was defined as arm lean mass [kg]/height [m]^2^. Leg SMI was defined as leg lean mass [kg]/height [m]^2^. Appendicular SMI was defined as the sum of arm and leg SMIs. Each participant held a digital hand dynamometer (Digital Grip Strength Dynamometer, T.K.K 5401, Takei Scientific Instruments Co., Ltd., Tokyo, Japan) in a sitting position. The maximum grip strength was measured with the elbow flexed at 90 degrees and the shoulder connected to the chest with the wrist at a neutral position (0 degrees) [17]. In this study, hand grip strength was used as a surrogate parameter to evaluate the function and predict the prognosis after hip fracture based on research results of Di Monaco et al. [18].

### Definition of osteoporosis

Osteoporosis was defined as a BMD 2.5 standard deviations (SDs) below the peak bone mass of a young, healthy, gender- and race-matched reference population according to the World Health Organization (WHO) diagnostic classification. BMD (T-score) was used to classify osteoporotic (T-score ≤ − 2.5), osteopenic (− 2.5 < T-score < − 1.0), and normal patients (T-score ≥ − 1).

### Hip-structure analysis (HSA)

To evaluate the hip-bone geometry, DXA scans were performed to analyze the femoral neck (FN), the intertrochanteric region (IT), and the femoral shaft (FS) using HSA program. The cross-sectional area (CSA), width (W), and cortical thickness (CT) were measured based on bone-mass profiles. The hip axis length (HAL) was measured along the femoral neck axis from the base of the greater trochanter to the inner pelvic brim. The femur axis length (FAL) was measured along the femoral neck axis from the base of the greater trochanter to the inner pelvic brim. We calculated the neck-shaft angle (NSA) as the angle between the neckline and a line through the shaft of the femur set by a Hologic software on the outer cortex of the femoral shaft below the region of interest [19–21].

Cross-sectional moment of inertia (CSMI, cm^4^) was calculated by the integral of the bone mass weighted by the square of the distance from the center of mass to indicate the bending stress in a cross section. Z-section modulus (Z, cm^3^) was the maximal bending strength of the cross-section estimated with Z = CSMI/y, where y was the maximum distance from the center of mass to the medial or lateral cortical margin [22].

### Statistical analyses

To select a control group, a propensity-score matching method was used. Factors considered to be important confounders affecting the occurrence of basicervical hip fractures were chosen for the propensity-score algorithm. A logistic model with basicervical hip fractures as the outcome and age, sex, weight, spinal T-score, hip T-score, and vitamin D levels as confounders was used to estimate propensity scores. To account for the matched design, we performed paired t-tests [23].

To compare means and proportions of each group, Student’s t-tests and Chi-squared (χ^2^) tests were conducted. Variables with *p*-values < 0.05 were included in the multivariate model.

A receiver operator curve analysis (ROC) was also performed to identify cutoff values for diagnosing basicervical hip fractures using HSA. All statistical tests were two-tailed and statistical significance was defined at *p* < 0.05. All statistical analyses were performed using SPSS Statistics V.22 (SPSS Inc., Chicago, IL, USA).

## Results

### Demographic characteristics of patients with basicervical hip fractures

Forty patients with femur neck fractures (FNF) and 19 patients with basicervical hip fractures (HF) were ultimately included in this study. Comparison of demographic data by the type of hip fractures revealed that age (*p* = 0.433), sex (*p* = 0.222), weight (*p* = 0.254), spinal T-score (*p* = 0.147), hip T-score (*p* = 0.603), skeletal muscle index (*p* = 0.303), and vitamin D level (*p* = 0.238) showed no statistically significant differences between the two groups. However, height and grip strength were significantly different between the two groups (Table 1).

**Table 1:** Demographic characteristics by presence of basicervical hip fracture

	FNF (*N* = 40)	Basicervical HF (*N* = 19)	*P*-value
Age (years)	77.38 ± 9.31	79.50 ± 9.48	0.433
Sex			0.222
Male	13 (32.5%)	3 (15.8%)	
Female	27 (67.5%)	16 (84.2%)	
Height (m)	160.45 ± 7.72	155.73 ± 6.16	0.015
Weight (kg)	55.48 ± 8.25	52.87 ± 8.02	0.254
Spine T-score	- 1.54 ± 1.40	−1.79 ± 1.65	0.147
Hip T-score	- 1.88 ± 0.82	− 1.99 ± 0.71	0.603
SMI (kg/m^2^)	5.71 ± 1.12	5.46 ± 0.68	0.303
Grip strength (kg)	15.37 ± 6.77	11.65 ± 4.92	0.021
VitD (ng/ml)	13.99 ± 11.05	17.96 ± 12.24	0.238

*FNF* Femur neck fracture, *HF* Hip fracture, *BMI* Body mass index, *SMI* Skeletal muscle index

### Demographic characteristics by the type of hip fracture after propensity score matching

Following propensity score matching, 19 patients in the FNF group and 19 patients in the basicervical HF group were ultimately included in this study. Comparison of their demographic data by the type of hip fracture showed that age (*p* = 0.579), sex (*p* = 1.00), weight (*p* = 0.311), spinal T-score (*p* = 0.957), hip T-score (*p* = 0.968), SMI (*p* = 0.231), and vitamin D level (*p* = 0.682) were not significantly different between the two groups (Table 2).

**Table 2:** Demographic characteristics by presence of basicervical hip fracture after Propensity score matching

	FNF (*N* = 19)	Basicervical HF (*N* = 19)	*P*-value
Age (years)	80.58 ± 6.59	79.11 ± 9.37	0.579
Sex			1.000
Male	4 (21.1%)	3 (15.8%)	
Female	15 (78.9%)	16 (84.2%)	
Height (cm)	159.87 ± 7.96	155.73 ± 6.16	0.082
Weight (kg)	55.79 ± 9.46	52.87 ± 8.02	0.311
Spine T-score	- 1.98 ± 1.39	−1.95 ± 1.62	0.957
Hip T-score	- 2.00 ± 0.89	−1.99 ± 0.71	0.968
SMI (kg/m^2^)	5.86 ± 1.25	5.46 ± 0.68	0.231
Grip strength (kg)	16.85 ± 6.78	11.65 ± 4.92	0.011
VitD (ng/ml)	16.17 ± 14.41	17.96 ± 12.24	0.682

*FNF* Femur neck fracture, *HF* Hip fracture, *BMI* Body mass index, *SMI* Skeletal muscle index

### Hip structural analysis by the presence of basicervical hip fracture

The cross-sectional moment of inertia **(**CSMI) of intertrochanter was significantly lower in patients with basicervical hip fractures (HF) than in patients with FNF (*p* = 0.045). However, no other HSA variables were significantly different between the two groups (Table 3).

**Table 3:** Hip structural analysis (HSA) by presence of basicervical hip fracture after Propensity score matching

	FNF (*N* = 19)	Basicervical HF (*N* = 19)	*P*-value
FAL (cm)	104.58 ± 7.31	101.74 ± 5.78	0.192
HAL (cm)	105.00 ± 6.18	101.95 ± 7.43	0.177
FN_CSA (cm^2^)	1.96 ± 0.41	1.95 ± 0.48	0.954
FN_CT (cm)	0.11 ± 0.03	0.11 ± 0.02	0.583
FN_Width (cm)	3.57 ± 0.37	3.43 ± 0.42	0.274
FN_CSMI (cm^4^)	1.96 ± 0.75	1.81 ± 0.97	0.595
ITN_CSA (cm^2^)	3.25 ± 0.83	2.86 ± 0.54	0.090
ITN_CT (cm)	0.25 ± 0.23	0.23 ± 0.04	0.171
ITN_Width (cm)	5.72 ± 0.65	5.45 ± 0.55	0.167
ITN_CSMI (cm^4^)	9.90 ± 4.20	7.57 ± 2.46	0.045
FS_CSA (cm^2^)	3.24 ± 0.72	3.02 ± 0.38	0.261
FS_CT (cm)	0.40 ± 0.08	0.38 ± 0.57	0.612
FS_Width (cm)	3.01 ± 0.32	2.91 ± 0.22	0.261
FS_CSMI (cm^4^)	2.99 ± 1.21	2.53 ± 0.57	0.137

*FNF* Femur neck fracture, *HF* Hip fracture, *FAL* Femur axis length, *HAL* Hip axis length, *CSA* Cross sectional area, *CT* Cortical thickness, *CSMI* Cross sectional moment of inertia, *FN* Femur neck, *ITN* Intertrochanter, *FS* Femur shaft

### ROC analysis for the diagnosis of basicervical HF using HSA

**Fig. 1:**
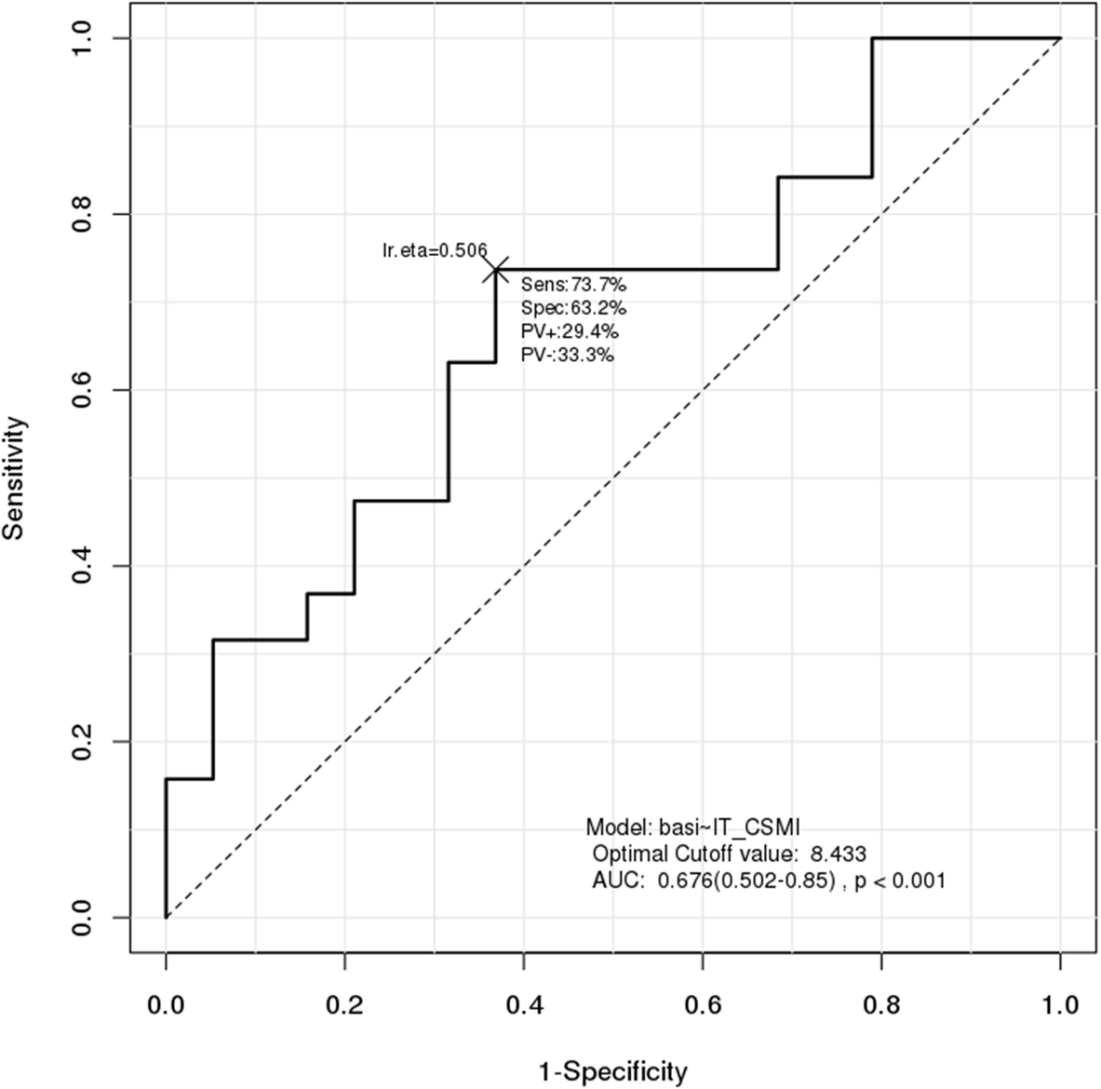
ROC analysis for the diagnosis of hip fractures using CSMI of the intertrochanter

**Fig. 2:**
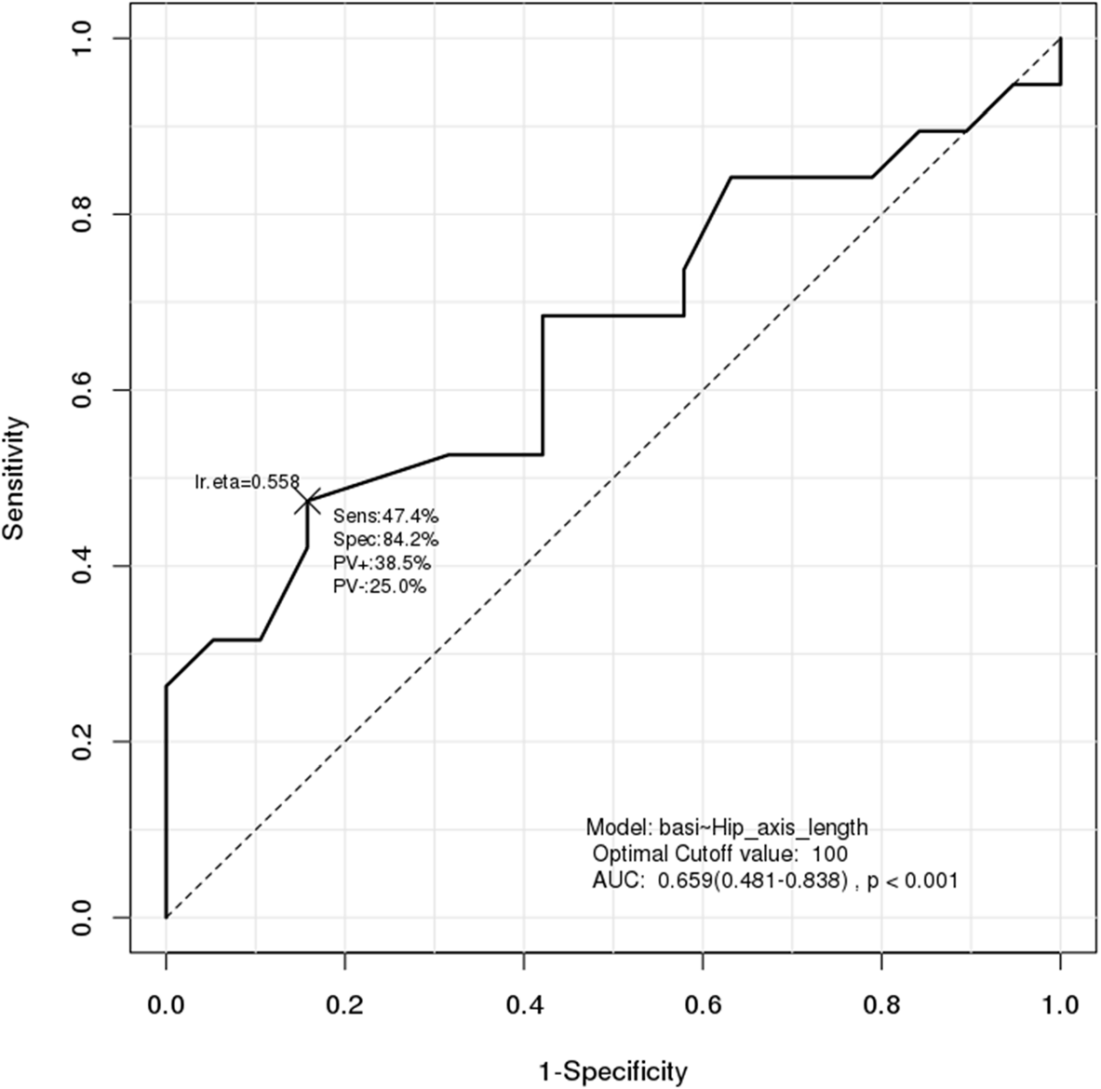
ROC analysis for the diagnosis of hip fractures using hip axis length

**Fig. 3:**
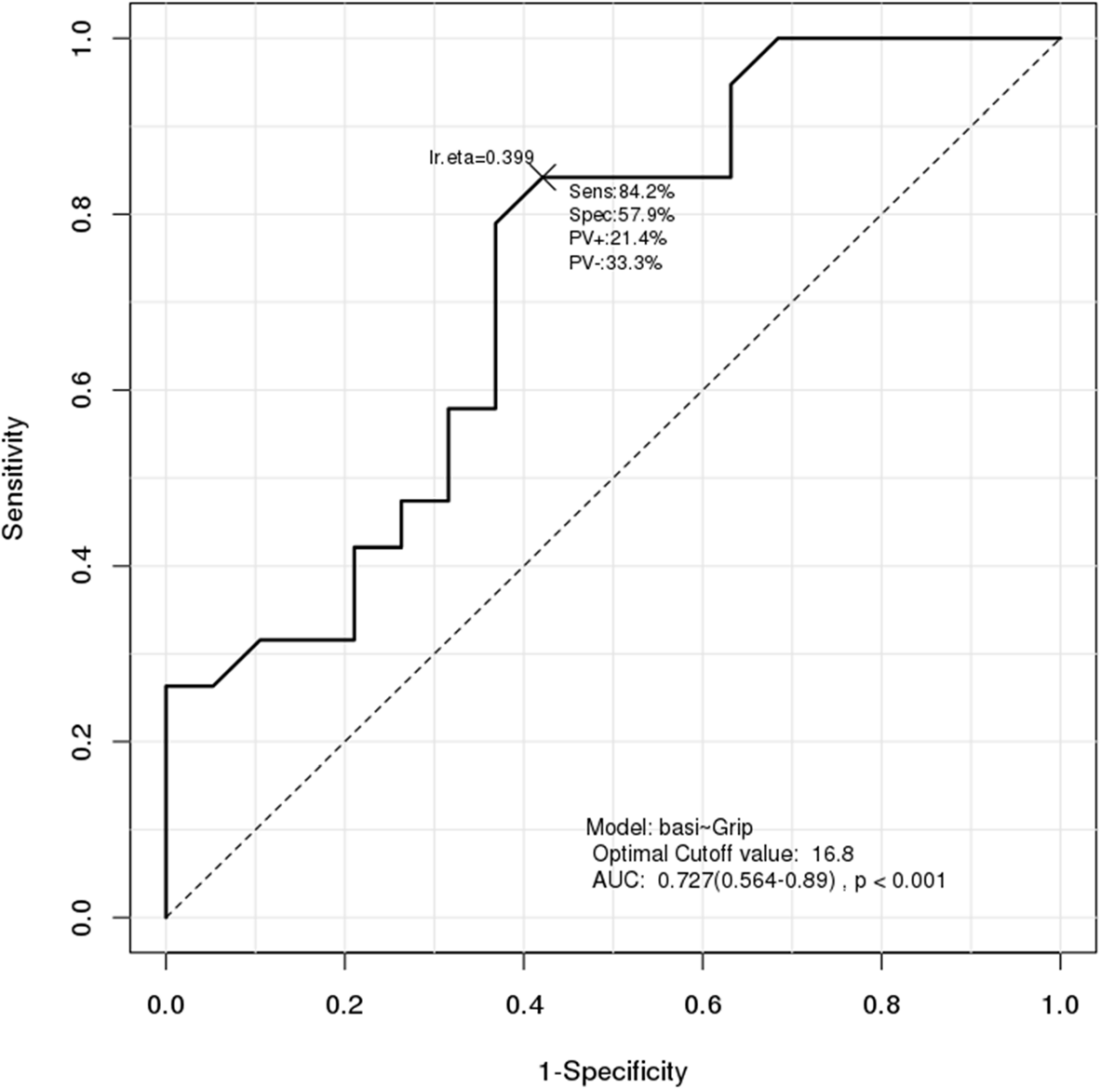
ROC analysis for the diagnosis of hip fractures using handgrip strength

**Fig. 4:**
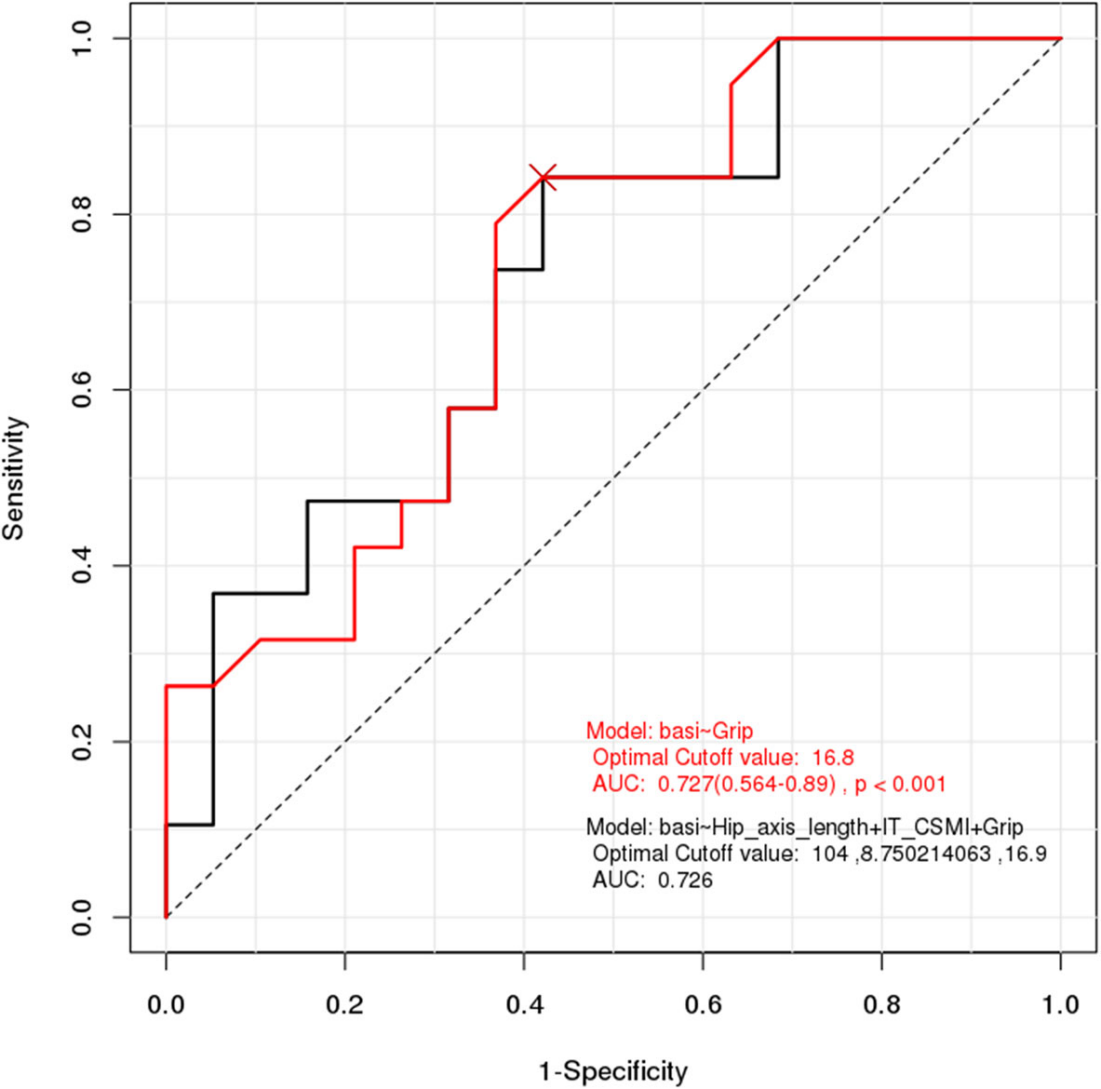
ROC analysis for the diagnosis of hip fractures using three variables

Results of ROC analysis showed that the cutoff point for HSA was 100 for HAL (AUC = 0.659, *p* < 0.001) and 5.712 for CSMI of the intertrochanter (AUC = 0.676, *p* < 0.001). ROC analysis showed that the cutoff point for handgrip strength was 16.8 kg (AUC = 0.727, *p* < 0.001) (Figs. 1, 2 and 3). Results of sensitivity and specificity are shown in Table 4. ROC analysis for HAL, CSMI of the intertrochanter, and handgrip strength showed cutoff points of 104.8, 8.75, and 16.9 (AUC = 0.726, *p* < 0.001), respectively (Fig. 4).

**Table 4:** ROC analysis for diagnosis of Basicervical hip fracture

Variables	Cut-off point	Sensitivity	Specificity	AUC	*P*-value
HAL	100	47.4%	84.2%	0.659	< 0.001
ITN_CSMI	8.433	73.7%	63.2%	0.676	< 0.001
HGS (kg)	16.8	84.2%	57.9%	0.727	< 0.001

*ROC* Receiver operating curve, *AUC* Area under the ROC Curve, *HAL* Hip axis length, *CSMI* Cross sectional moment of inertia, *ITN* Intertrochanter, *HGS* Hand grip strength

## Discussion

BMD can predict osteoporotic hip fractures relatively well except for fractures caused by high energy trauma [24]. However, it does not explain differences that exist between these fractures occurring in the vicinity of the proximal femur region. Fractures occur when the external force exceeds the bone’s ability to absorb energy through combined effects of elastic and plastic deformation [15]. Decreases in bone density due to aging and osteoporosis are accompanied by geometrical changes, which alter bones’ ability to absorb energy and resist fractures. These changes cannot be explained simply by BMD. They can be determined through HSA [16].

Bending and axial compression forces are applied to the proximal femoral area when an elderly person falls [15]. In a previous comparative study of HSA between hip fracture patients and non-fracture patients, hip fracture patients were found to have lower bending strength (section modulus), lower axial strength (coronal sectional area), thinner cortex, and wider diameter [15]. The present study also found that HSA parameters were more consistent with intertrochanteric fractures than with femoral neck fractures. We hypothesized that there were differences in HSA parameters between these fractures occurring at close anatomical locations. The main finding of this study was that the CSMI was significantly lower in basicervical fractures. The CSMI is used to measure the distribution of material around the neck axis necessary to calculate the resistance to bending. The mechanical stress within a cross-section subjected to bending is inversely related to the CSMI. It varies with the distance from the neutral axis [25, 26]. In other words, basicervical fractures have a lower bending strength than femoral neck fractures, which occur near the intertrochanteric line.

Lower CSMIs in basicervical fractures can have clinical significance from two perspectives. The first is the importance in terms of diagnostic prediction. Several HSA studies on the prediction of various hip fracture incidences have reported that various HSA parameters are important, including bone cross-sectional area, outer diameter, selection modulus, buckling ratio, the HAL, and the strength index [14, 27]. Among them, the section modulus is a strength parameter derived from the CSMI. It is equal to the CSMI divided by the centroidal distance, the distance from the centroidal axis to the edge of the section [16]. Also, in a study by Kaptoge et al. [15], BMD, CSA, the CSMI, section modulus, and the cortices in the hip fracture group are lower than those in the fracture-free group. Resistance to bending strength is important for the prediction of hip fracture occurrence. Moreover, the CSMI, an indicator of bending strength, can be used as an index to predict the location of hip fractures. The second is the importance of therapeutic aspects of fractures. Various studies have reported a high rate of treatment failure for basicervical fractures [4, 7, 9, 10]. These studies noted that morphological characteristics that tended to collapse into the intertrochanteric area were due to their anatomical locations and identified them as major causes of treatment failure. However, our study showed that weakness in bending strength was another important factor to consider when selecting surgical treatment for basicervical fractures. Sharma et al. [28] have reported that multiple cancellous screws have a higher failure rate than dynamic hip screw and nail in the treatment of basicervical neck fractures and argued that multiple cancellous screws should not be used for such fractures. Imren et al. [29] have also reported that fixed angle devices have higher fixation strengths than multiple cancellous screws in a biomechanical study comparing treatment methods for basicervical neck fractures. Therefore, we believe that results of this study provide evidence that fixed angle devices should be used in the selection of surgical methods for such fractures.

Leslie et al. [30] have hypothesized that different types of bone geometry can predict fractures. In the Fracture Risk Prediction Model using DXA-based finite element analysis, a study has reported that the HAL and strength index are significantly associated with the incidence of hip fracture [30]. In a study by Faulkner et al. [31], the HAL of the hip fracture group is also significantly shorter than that of the control group, indicating a reduced capacity to withstand a fall. They have identified HAL as an independent predictor of hip fractures. They reported that an increase in HAL equivalents to 1 SD was associated with a 1.8-fold increase in the risk of hip fractures in women [30]. Results of the present study showed that the average HAL of patients with basicervical fracturse was shorter than that of patients with femoral neck fractures. The diagnostic significance of basicervical fractures was found in the ROC analysis. Thus, HAL is not only a predictor of fracture risk, but also a significant predictor of fracture type.

The European Working Group on Sarcopenia in Older People has developed a clinical definition of sarcopenia based on low muscle mass and reduced muscle function [32]. Grip strength is recommended as a good simple measure of muscle strength. In older people, falls are associated with lower physical activity, which is one of signs of reduced physical function [33, 34]. Sarcopenia is also closely related to osteoporosis and osteoporotic hip fractures. A study by Travison et al. [35] on the correlation between body mass index and proximal femur strength has reported a fracture prevention effect in people with high BMI, not because of fat, but because of muscle mass. Our study confirmed that handgrip strength was decreased statistically in the basicervical fracture group and that handgrip strength was a significant predictor for basicervical fracture diagnosis. We do not know the exact mechanism to explain this result. It might affect the loading occurring during falls or the absorption of impact force. Therefore, further studies are needed.

This study has several limitations. First, the study sample size was small. However, we used propensity score matching to address this problem. Second, the study design was retrospective. We could not exclude the possibility of selection bias. Third, we did not consider comorbidity or the use of anti-osteoporotic medications by patients. However, we tried to complement this by analyzing factors such as skeletal muscle index, grip strength, and vitamin D levels. Fourth, we believe that the severity of injury, the mechanism of injury, and leg position at the time of injury could affect the occurrence of fracture. However, these factors could not be reflected in our study.

In conclusion, proximal femoral geometric analysis using HSA is a useful method for predicting the type of hip fracture. Additionally, lower CSMI, shorter HAL, and lower grip strength are major predictors of basicervical fractures.

## Data Availability

All data generated or analysed during this study are included in this article.

## Abbreviations

DHS: Dynamic hip screws
CMN: Cephallomedullary nailing
HSA: Hip structural analysis
FNF: Forty patients with femur neck fractures
HF: Hip fractures
BMD: Bone mineral density
SMI: Skeletal muscle index
SDs: Standard deviations
WHO: World Health Organization
FN: Femoral neck
IT: Intertrochanteric region
FS: Femoral shaft
CSA: Cross-sectional area
W: Width
CT: Cortical thickness
HAL: Hip axis length
FAL: Femur axis length
NSA: Neck-shaft angle
